# Stroke as the Leading Cause of Death in Rural Ludhiana, India: A Verbal Autopsy From the Population-Based Rural Stroke Registry of Ludhiana

**DOI:** 10.7759/cureus.103592

**Published:** 2026-02-14

**Authors:** Shavinder Singh, Clarence J Samuel, Deepshikha Kamra, Melkey S Bunyan, Ranjit Injety, Jeyaraj D Pandian

**Affiliations:** 1 Department of Community Medicine, Christian Medical College and Hospital, Ludhiana, IND; 2 Department of Neurology, Christian Medical College Ludhiana, Ludhiana, IND; 3 Department of Neurology, Christian Medical College and Hospital, Ludhiana, IND

**Keywords:** cause of death, epidemiology and public health, hypertension, rural india, stroke, stroke mortality, verbal autopsy

## Abstract

Introduction

Stroke is the second leading cause of global mortality, with a disproportionately high burden in low- and middle-income countries (LMICs). In regions with limited medical certification of death, verbal autopsy (VA) serves as a critical tool for determining the cause-of-death distribution. This study aimed to determine the stroke mortality in rural Ludhiana, Punjab, India.

Materials and methods

A prospective, population-based study was conducted between December 2016 and November 2018 across two rural blocks in Ludhiana (Pakhowal and Sidhwan Bet), covering a total population of 236,266. Frontline health workers identified all-cause mortality, followed by home-based VA. To ensure data integrity and minimize misclassification, two independent neurologists adjudicated stroke deaths using World Health Organization (WHO) standards and Indian Council of Medical Research (ICMR) VA tools, with a third expert resolving discrepancies. We cross-referenced registry data with municipal corporation and maintained consistency through monthly field audits and random interview re-evaluations. Cause-specific mortality fractions (CSMF), crude stroke mortality rates (CSMR), and age-standardized mortality rates (ASMR) were calculated.

Results

A total of 1,182 deaths were recorded, of which stroke was identified as the leading cause, accounting for 262 (22.2%) deaths. This represents a CSMF of 22.2% (95% confidence interval {CI}: 19.8-24.5). The annualized crude stroke mortality rate was 55.4 per 100,000 (95% CI: 48.9-62.5). While men had a higher crude rate (57.4), women exhibited a higher CSMF (24.7% versus 20.5%). After adjusting for the age distribution of the population using the WHO World Standard, the annualized ASMR was 29.7 per 100,000 person-years (95% CI: 26.1-33.4). Hypertension (214, 81.7%) and diabetes (89, 34.0%) were the most prevalent risk factors. Alarmingly, 219 (83.6%) of stroke deaths occurred within 30 days of symptom onset, with 126 (48.1%) occurring within the first seven days.

Conclusion

Stroke has emerged as the leading cause of death in rural Ludhiana, accounting for 262 (22.2%) of all mortality. While VA-based diagnosis carries inherent recall limitations, our rigorous adjudication protocol provides a reliable estimate of this burden. The high acute mortality rate, coupled with a 214 (81.7%) prevalence of uncontrolled hypertension, reveals critical gaps in both primary prevention and hyperacute care. These findings highlight an urgent need for integrated public health strategies, specifically focusing on community awareness and the implementation of "hub-and-spoke" acute care pathways to mitigate this rising epidemic.

## Introduction

Globally, stroke is the second leading cause of death and the third leading cause of disability. Data from the low- and middle-income countries (LMICs) show a disproportionately high burden, with age-standardized mortality rates (ASMR) and disability-adjusted life years (DALYs) exceeding the global average by a factor of four. One in four adults over 25 will have a stroke in their lifetime [1,2].

The Southeast Asia Region (SEAR) makes up more than 40% of the world's stroke mortality and covers 11 culturally, geographically, and socially varied nations that are transitioning to noncommunicable diseases. Stroke care throughout all stages varies significantly among SEAR nations, despite common issues in healthcare access, awareness, and patient behavior. There is a paucity of high-quality stroke epidemiology data from this region. Although ischemic stroke is common, intracerebral hemorrhage is more common in several SEAR countries than in the rest of the world [3]. A number of factors contribute to this high burden, including an aging population, rising risk factors (diabetes, hypertension, alcohol and tobacco use, and physical inactivity), and environmental issues, including air pollution and extreme weather conditions [4].

According to the Indian Global Burden of Disease (GBD) Study 1990-2019, stroke is the main contributor to neurological-related mortality and the largest contributor to DALYs [5]. Studies across various regions, including Mumbai, Trivandrum, Ludhiana, Kolkata, and rural West Bengal, reveal a high incidence of stroke, with high case fatality and mortality rates. The one-month case fatality was between 18% and 42% [6]. According to the Indian Council of Medical Research (ICMR)-National Centre for Disease Informatics and Research (NCDIR) report, the crude incidence of stroke was 138.1 per 100,000 population. Age-standardized case fatality rates per 100,000 population for the pooled population-based stroke registry (PBSR) per 100,000 were 20.0 for men and 18.8 for women. In Cuttack, Cachar, and Tirunelveli PBSR, the percentage of registered cases from rural regions (ranging from 57.2% to 85.2%) exceeded that from urban areas (ranging from 14.8% to 42.8%), attributable to the substantial rural population [7]. A study on stroke incidence in urban Kolkata, North India, revealed a standardized incidence rate of 145.3 per 100,000 persons per year and a 30-day case fatality rate of 41%, both above estimates from industrialized countries [8]. A study conducted in urban and rural regions of Ludhiana compared stroke profiles and outcomes, where hemorrhagic stroke was seen more in rural regions than in urban regions. Age-standardized incidence rates in the rural area were 218.5/100,000 in 2017 and 197.6/100,000 in 2018, with drug abuse and hypertension being the most common risk factors for stroke [9,10].

According to the National Stroke Registry Programme (NSRP), ICMR-NCDIR shows that among all the registries, Varanasi PBSR reported the highest case fatality rate (CFR) (46.6 per 100,000 population) owing to stroke, followed by Cachar PBSR (39.9) and Cuttack PBSR (31.4). The case fatality proportion varied from 12.2% in Kota PBSR to 41.2% in Cachar PBSR. Kota PBSR had the lowest crude mortality rate (CMR) per 100,000 people (16.2), while the Varanasi registry had the highest (48.9). Among all the registries, Kota PBSR had the lowest age-adjusted case fatality rate per 100,000 people (13.0), whereas Varanasi PBSR had the highest (35.5) [11]. Chronic illnesses were the main cause of death in a study on the causes of death in rural Andhra Pradesh, South India. The majority of deaths were caused by circulatory system diseases (32%), with similar percentages of ischemic heart disease (14%) and cerebrovascular disease (13%) [12]. A study in the Gadchiroli district documented stroke as the leading cause of death, accounting for 14.3% of mortalities [13].

Despite the documented rise of stroke across India, significant disparities persist between urban centers and rural agrarian communities. Current national data often overlook the specific challenges of rural North India, where an aging population and high hypertension prevalence coincide with limited acute care infrastructure. While urban registries provide clinical insights, a critical knowledge gap remains regarding the precise, annualized stroke mortality burden in these underserved regions. Stroke deaths are often underestimated due to misclassification in mortality data documentation. To address this critical issue, a multipronged approach is needed. To overcome these challenges in LMICs with limited resources, verbal autopsy (VA) emerges as a reliable alternative method to assess mortality at a population level [14]. The VA tool records the description of the symptoms and the circumstances leading to death. The data are analyzed manually by a physician who assigns an International Classification of Diseases, Tenth Revision (ICD-10) code for the underlying cause of death based on clinical guidelines [14,15]. Hence, we aimed to determine mortality due to stroke by using verbal autopsy to demonstrate the cause of death as a part of the Ludhiana rural population-based stroke registry.

## Materials and methods

This investigation was conducted as part of phase II of the population-based rural stroke registry established in Ludhiana, Punjab. The study was implemented in two designated rural administrative blocks of the Ludhiana district: Pakhowal and Sidhwan Bet. A standardized verbal autopsy procedure was incorporated as a core component of the study protocol to ascertain stroke-related mortality and related epidemiological parameters. Ethical permission was obtained from the Institutional Ethics Committee of Christian Medical College Ludhiana (approval number: MCL-IEC/2013/0171/APPRVL/NEUROLOGY-ICMR), and the project was funded by the Task Force of ICMR.

After receiving training, accredited social health activists (ASHAs) were able to identify stroke victims in the community, who were then assessed by a neurologist [10]. The stroke cases were followed up by two research teams at home or at the hospital where they were admitted. The Ludhiana population-based stroke registry used verbal autopsy (VA) methods to collect data on stroke mortality, especially for deaths occurring at home where medical certification is unavailable. The VA tool (see Appendices) used was developed according to the World Health Organization (WHO) and Indian Council of Medical Research (ICMR) methodologies, and their feasibility was evaluated in a 2015 study [16]. To ensure data integrity, we implemented a multistage quality control process. We cross-referenced the registry and mortality data (hospital and home deaths) with municipal corporation centers to maximize capture rates. Two independent physicians reviewed all verbal autopsy narratives to confirm the cause of death, with a third expert resolving any discrepancies. Additionally, monthly field audits and a random review of interviews ensured data consistency. Individual training sessions and workshops were held in Ludhiana for the research staff and representatives of ICMR on both theoretical and practical aspects during the workshop.

**Figure 1:**
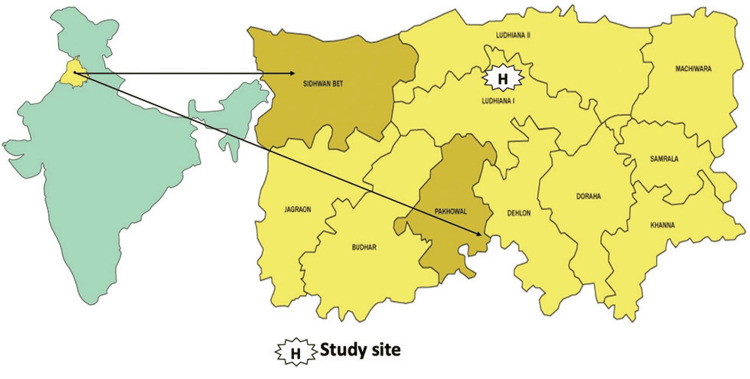
Map of Block Pakhowal and Sidhwan Bet Note: Image sourced from "Multicomponent short-term training of ASHAs for stroke risk factor management in rural India," by Jain M, Pandian J, Samuel C, Singh S, Kamra D, Kate M, 2019, J Neurosci Rural Pract, 10(4), 592-8 [18] under Creative Commons Attribution-NonDerivative-NonCommercial License (https://creativecommons.org/licenses/by-nc-nd/4.0/). No changes were made

This research was conducted across more than 150 villages in the rural blocks of Pakhowal and Sidhwan Bet, located in the Ludhiana district (Figure 1). According to 2011 census figures, the populations of Pakhowal and Sidhwan Bet were 109,201 and 127,065, respectively. The study's inclusion criteria focused on incident (first-ever) stroke cases and stroke-related fatalities identified during a two-year screening period from December 1, 2016, to November 30, 2018 [17,18].

Research teams visited deceased patients' homes and additional village households daily (at least five) to collect data using a verbal autopsy questionnaire. Local health workers provided death lists. Collected data was reviewed for errors by investigators and neurologists, who ultimately determined the stroke-related deaths. The study was conducted over a 24-month (two-year) surveillance period. To calculate the mortality rates, the total population at risk (236,266) was multiplied by the duration of the study, resulting in 472,532 person-years. To determine the stroke mortality burden in rural Ludhiana, we calculated the cause-specific mortality fraction (CSMF) as the proportion of stroke deaths relative to all-cause mortality, including 95% confidence intervals (CI). We expressed crude stroke mortality rates (CSMR) per 100,000 person-years. To enable global comparisons, we calculated age-standardized mortality rates (ASMR) using the WHO World Standard Population. We analyzed categorical variables, including sex-stratified risk factor prevalence (hypertension, diabetes, heart disease, and smoking) and time-to-death intervals (<7 days, ≤30 days, and >30 days). Finally, we reported mean ages at death with standard deviations (SD).

## Results

The analysis of the data revealed that out of 1,182 total deaths recorded (Table 1), stroke was the leading cause of mortality, accounting for 262 (22.2%) deaths. This represents a cause-specific mortality fraction (CSMF) of 22.2% (95% CI: 19.8-24.5) (Table 1). The annualized crude stroke mortality rate for the combined census population was 55.4 per 100,000 (95% CI: 48.9-62.5) (Table 1). While the crude rate was higher in men (57.4) than in women (53.2), the CSMF was notably higher in women (24.7%) compared to men (20.5%) (Table 1).

**Table 1:** Total and Stroke Mortality (N=262) CI: confidence interval

Variables	Male	Female	Total
Total deaths (n)	708	474	1,182
two-year population person-years	252,646	219,886	472,532
Stroke deaths, n (%)	145 (55.3%)	117 (44.7%)	262 (100%)
Cause-specific mortality fraction (CSMF), % (95% CI)	20.5% (17.5-23.5)	24.7% (20.8-28.6)	22.2% (19.8-24.5)
Mean age of stroke deaths (SD)	69.0 (15.0)	73.1 (12.2)	70.8 (13.9)
Crude stroke mortality rate per 100,000 (95% CI)	57.4 (48.5-67.4)	53.2 (44.0-63.7)	55.4 (48.9-62.5)

**Figure 2:**
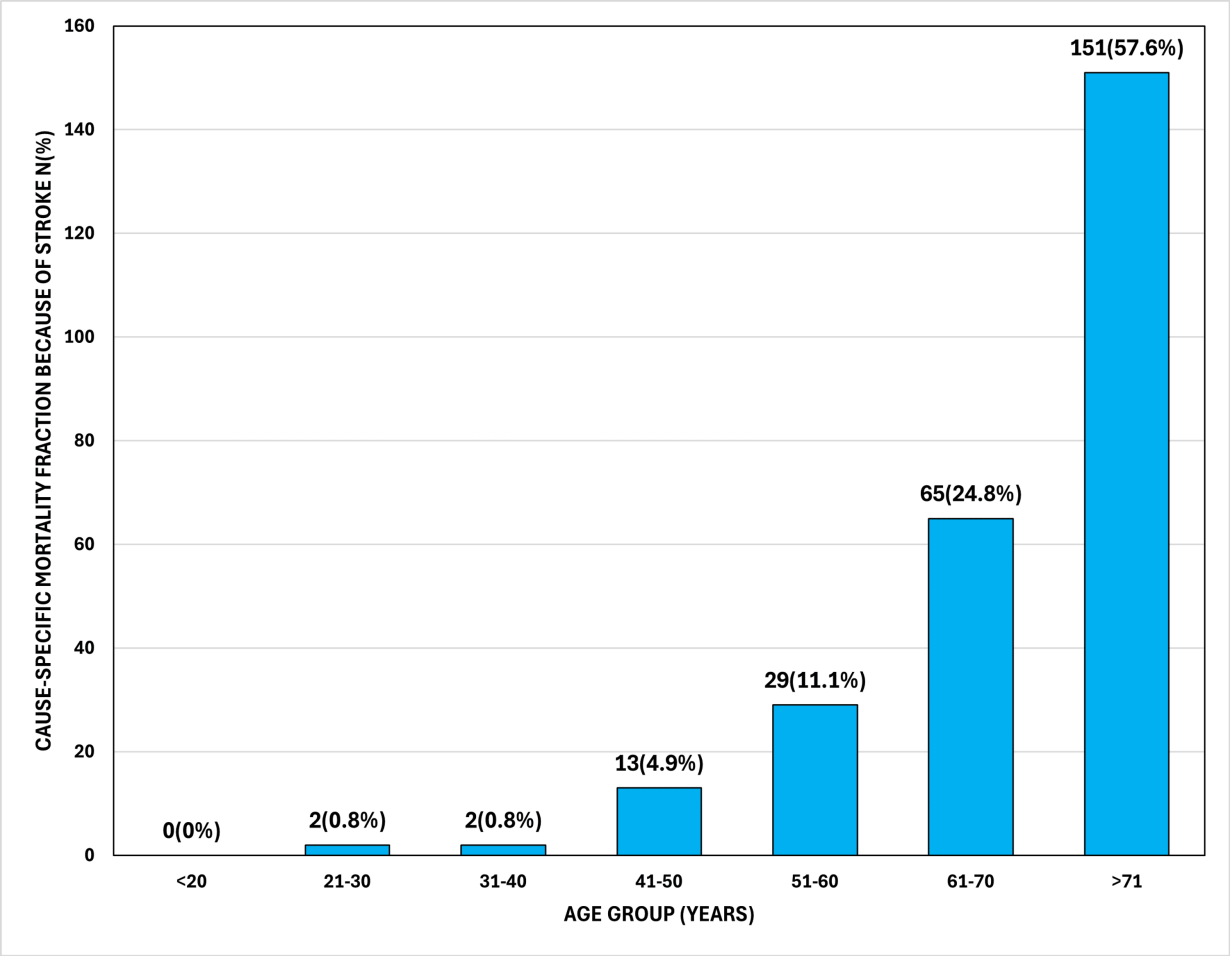
Age-Wise Cause-Specific Mortality Fraction Because of Stroke (N=262)

The mean age of death due to stroke was 70.8 years, with women dying at a significantly higher mean age (73.1±12.2 years) than men (69.0±15.0 years) (Table 1). Age-specific crude rates showed an exponential increase with age (Table 2), with the highest mortality observed in the ≥85 age group at 141.7 per 100,000 (Table 2). Distribution analysis (Figure 2) showed that 151 (57.6%) of all stroke deaths occurred in individuals aged 71 years and older.

**Table 2:** Age-Wise Crude Stroke Mortality Rates Crude stroke mortality rate/100,000 person-years

Age Group (Years)	Women Rate	Men Rate	Total Rate (Annualized)
<25	0.0	5.6	3.0
25-29	0.0	5.5	3.0
30-34	0.0	5.5	3.0
35-39	0.0	5.5	2.9
40-44	6.4	11.0	8.8
45-49	19.1	38.8	29.4
50-54	12.7	60.7	38.4
55-59	25.4	66.2	47.2
60-64	82.6	82.8	82.7
65-69	139.7	82.7	109.2
70-74	133.5	138.0	135.9
75-79	82.6	71.8	76.8
80-84	95.4	88.3	91.5
≥85	146.1	137.9	141.7
Total (crude)	53.2	57.4	55.4

Hypertension was the most prevalent risk factor among stroke fatalities, present in 214 (81.7%) of cases, followed by diabetes mellitus at 89 (34.0%). It has been observed that the prevalence of hypertension (99, 84.6%) and diabetes (45, 38.5%) is higher in women (Table 3).

**Table 3:** Prevalence of Risk Factors for Stroke (N=262)

Risk Factors	Male, n (%)	Female, n (%)	Total, n (%)
Hypertension	115 (79.3%)	99 (84.6%)	214 (81.7%)
Diabetes mellitus	44 (30.3%)	45 (38.5%)	89 (34.0%)
Heart disease	28 (19.3%)	17 (14.5%)	45 (17.2%)
Smoking	22 (15.2%)	0 (0.0%)	22 (8.4%)

Regarding the clinical timeline, the majority of deaths were acute; 219 (83.6%) of deaths occurred within 30 days of symptom onset, with 126 (48.1%) occurring within the first seven days (Table 4).

**Table 4:** Time to Death From the Onset of Symptoms of Stroke

Time to Death From the Onset of Symptoms, n (%)	Women (n=117)	Men (n=145)	Total (n=262)
≤30 days	92 (78.6%)	127 (87.6%)	219 (83.6%)
<7 days (subset)	50 (42.7%)	76 (52.4%)	126 (48.1%)
>30 days	12 (10.3%)	12 (8.3%)	24 (9.2%)
Unclear from the narrative	13 (11.1%)	6 (4.1%)	19 (7.3%)

The annualized age-standardized mortality rate (ASMR) was 29.7 per 100,000 person-years (95% CI: 26.1-33.4) (Table 5).

**Table 5:** Annualized Age-Standardized Mortality Rate (ASMR) Rates per 100,000 person-years WHO: World Health Organization

Age Group	Two-Year Deaths	Population	WHO Weight	Weighted Rate
<25	1	116,561	0.4284	0.18
25-29	1	16,910	0.0793	0.23
30-34	1	17,043	0.0761	0.22
35-39	1	17,212	0.0715	0.21
40-44	3	17,015	0.0659	0.58
45-49	10	13,634	0.0604	2.22
50-54	13	11,728	0.0537	2.98
55-59	16	11,664	0.0455	3.12
60-64	28	12,692	0.0372	4.10
65-69	37	11,446	0.0296	4.78
70-74	46	11,043	0.0221	4.60
75-79	26	9,771	0.0152	2.02
80-84	45	8,203	0.0091	2.50
≥85	34	5,294	0.0063	2.02
Total	262	236,266	1.000	29.7

## Discussion

Stroke represents a significant public health burden, often resulting from thrombotic occlusion within the cerebral vasculature [19]. The results of this community-based mortality registry in rural Ludhiana demonstrate that stroke is the preeminent cause of death, accounting for 22.2% of all recorded fatalities. This finding is significantly higher than the results reported by Kalkonde et al. in the Gadchiroli study, which found that stroke was the leading cause of death in rural Maharashtra, accounting for 14.3% of all deaths [13]. While both studies identify stroke as a primary public health threat, the higher cause-specific mortality fraction (CSMF) in Ludhiana (22.2%) suggests an even more advanced epidemiological transition in rural Punjab compared to rural Gadchiroli. This disparity likely reflects regional differences in lifestyle, dietary patterns, and the prevalence of metabolic risk factors [20].

The demographic profile of stroke mortality in our study also shows a distinct pattern. While the annualized crude mortality rate was higher in men (57.4 per 100,000) than in women (53.2 per 100,000), women exhibited a higher CSMF (24.7% versus 20.5%), indicating that stroke is a more dominant cause of death within the female cohort. Furthermore, the mean age of stroke death for women (73.1 years) was significantly higher than for men (69.0 years). This mirrors observations by Kalkonde et al., where stroke was a major killer across both sexes, but emphasizes that in Ludhiana, stroke mortality is heavily concentrated in the geriatric population, with 57.4% of deaths occurring in those aged 71 and above [13].

A critical finding in our registry is the high acute mortality rate, with 219 (83.6%) of deaths occurring within 30 days and 126 (48.1%) within the first seven days. This "acute-heavy" mortality profile is even more pronounced than that seen in other Indian verbal autopsy studies and highlights a severe lack of access to specialized acute care [21,22]. The overwhelming presence of hypertension (214, 81.7%) as the leading risk factor in our cohort reinforces the conclusion by Kalkonde et al. [13] that stroke is the "new epidemic" in rural India, driven primarily by uncontrolled blood pressure in communities with limited healthcare access [23].

From a public health perspective, these findings necessitate a multi-level intervention strategy focused on prevention, early recognition, and infrastructure development. Given that over 80% of stroke victims were hypertensive, we should intensify efforts through the India Hypertension Control Initiative (IHCI) to ensure a steady supply of antihypertensive medications at primary health centers (PHCs) and improve patient adherence [24]. To address the 126 (48.1%) deaths occurring in the first week, we should launch community awareness campaigns using Face, Arm, Speech, and Time (FAST), training community health workers (ASHAs and auxiliary nurse midwives {ANMs}) to educate families on symptom recognition to reduce prehospital delays [25]. Furthermore, Ludhiana is strengthening its "hub-and-spoke" model by equipping tertiary centers with dedicated stroke units while training rural PHCs to stabilize and rapidly transfer patients, as Tezpur has adopted [26]. In remote blocks such as Sidhwan Bet and Pakhowal, tele-consultation services are being explored to allow rural clinicians to consult with neurologists in real time, facilitating earlier diagnosis and treatment initiation before a patient reaches a tertiary facility [27].

Study strengths and limitations

The major strength of this study is its prospective, community-based design covering a large census population of over 236,000 individuals, which minimizes the selection bias often found in hospital-based registries. The use of physician-adjudicated verbal autopsy provides a reliable estimate of mortality in a region where formal medical certification of death is often unavailable. However, this study has certain limitations. The reliance on verbal autopsy involves inherent recall bias regarding symptoms and risk factors. To minimize the resulting risk of misclassification, we employed a rigorous quality control framework, including independent dual-physician adjudication and standardized WHO-ICMR protocols. Additionally, as a mortality registry, it does not capture nonfatal strokes, which means the total "stroke burden" (including disability) is likely much higher than reported here.

## Conclusions

This community-based mortality registry confirms that stroke has become the leading cause of death in rural Ludhiana, accounting for nearly one-quarter of all fatalities. The high cause-specific mortality fraction (22.2%) and the stark 30-day mortality rate (83.6%) highlight a critical public health crisis in rural Punjab. These findings suggest that the region is experiencing an advanced epidemiological transition, where the burden of vascular disease now surpasses traditional infectious etiologies. The overwhelming prevalence of hypertension among stroke victims underscores the urgent need for robust primary prevention and grassroots blood pressure control programs, such as the India Hypertension Control Initiative (IHCI). Furthermore, the high rate of early mortality within the first week of symptom onset points to a significant gap in acute care access and community awareness.

To mitigate this burden, a comprehensive strategy integrating and expanding the FAST acronym for early symptom recognition, a decentralized "hub-and-spoke" model for acute care, and the expansion of tele-stroke services are essential. Addressing these systemic gaps is vital to reducing stroke-related mortality and improving the survival outcomes of rural populations in India.

## Appendices

Table 6 shows the VA form. 

**Table 6:** ICMR Population-Based Stroke Registry Verbal Autopsy Form ICMR: Indian Council of Medical Research

Serial number	Questions	Options	
1	Name		
2	Age (completed years)		
3	Sex	Male/female	
4	Address		
5	Date of death		
6	Date of verbal autopsy		
7	Occupations of the deceased		
8	Informant's name		
9	Relation to the dead	Mother/father/son/daughter/others	
10	Was the informant present at the time of the death of the person?	Yes/no	
11	Did the informant live with the deceased?	Yes/no	
12	Where did the death occur?	Hospital/home/elsewhere	
	Details of death and the events before the death		
13	Was there a history of risk factors?	Hypertension	Yes/no
		Diabetes mellitus	Yes/no
		Smoking	Yes/no
		Heart disease	Yes/no
14	Detailed verbal autopsy of the cause of death (details of previous illness, treatment, and the events leading to death)		
	Was the deceased ill prior to death?	Yes/no	
	Did she/he have weakness on one side of the body prior to death?	Yes/no	
	Did that weakness develop suddenly?	Yes/no	
	Did it last more than 24 hours?	Yes/no	
	Was it a sudden death?	Yes/no	
	Was there a history of severe headache just prior to death?	Yes/no	
	How many days was the patient ill before death?		
	Was the patient seen by a medical or health professional?	Yes/no	
	Was the patient admitted to a hospital or a clinic?	Yes/no	
	How many nights?		
15	What is the presumed cause of death?		
16	Name of the investigator completing the form		
